# Impact of adherence to cancer-specific prevention recommendations on subsequent risk of cancer in participants in Alberta’s Tomorrow Project

**DOI:** 10.1017/S1368980018002689

**Published:** 2018-10-22

**Authors:** Jian-Yi Xu, Jennifer E Vena, Heather K Whelan, Paula J Robson

**Affiliations:** 1 Alberta’s Tomorrow Project, CancerControl Alberta, Alberta Health Services, Richmond Road Diagnostic and Treatment Centre, 1820 Richmond Road SW, Calgary, AB, Canada, T2T 5C7; 2 Department of Health and Physical Education, Faculty of Health, Community and Education, Mount Royal University, Calgary, AB, Canada; 3 Department of Agricultural, Food and Nutritional Science, Faculty of Agricultural, Life and Environmental Science, University of Alberta, Edmonton, AB, Canada

**Keywords:** Cancer prevention recommendations, Cancer risk, Cohort study, Lifestyle factors, Public health

## Abstract

**Objective:**

The World Cancer Research Fund (WCRF) and the American Institute for Cancer Research (AICR) publish recommendations for cancer prevention. The present study aimed to estimate the association between adherence to these cancer-specific prevention recommendations and subsequent development of cancer in a prospective cohort.

**Design:**

A composite adherence score was constructed based on questionnaire data to reflect overall adherence to WCRF/AICR lifestyle-related recommendations on body fatness, physical activity, diet and alcoholic drinks. Multivariable Cox proportional hazard regression models were used to assess the association (hazard ratio; 95 % CI) between the adherence score and risk of developing cancer.

**Setting:**

Alberta’s Tomorrow Project, a prospective cohort study.

**Participants:**

Men and women (*n* 25 100, mean age at enrolment 50·5 years) recruited between 2001 and 2009 with no previous cancer diagnosis were included in analyses.

**Results:**

Cancer cases (*n* 2066) were identified during a mean follow-up of 11·7 years. Participants who were most adherent to the selected WCRF/AICR recommendations (composite score: 4–6) were 13 % (0·87; 0·78, 0·98) less likely to develop cancer compared with those who were least adherent (composite score: 0–2). Each additional recommendation met corresponded to a 5 % (0·95; 0·91, 0·99) reduction in risk of developing cancer. When stratified by sex, the associations remained significant for women, but not for men.

**Conclusions:**

Adherence to lifestyle-related cancer prevention recommendations was associated with reduced risk of developing cancer over the follow-up term in this Canadian cohort.

According to the GLOBOCAN estimates for the International Agency for Research on Cancer, there were 14·1 million new cancer cases and 8·2 million cancer-related deaths in 2012^(^ ^1^ ^,^ ^2^ ^)^. However, individual studies and meta-analyses suggest a large proportion of cancer cases may be prevented through management of modifiable lifestyle factors such as tobacco exposure, excess alcohol consumption, unhealthy diet, excess body weight and physical inactivity^(^ ^3^ ^,^ ^4^ ^)^. As a result, in 2007 the World Cancer Research Fund (WCRF) and the American Institute for Cancer Research (AICR) published evidence-based recommendations on food and nutrition, physical activity, body composition and other modifiable factors with the aim of reducing the risk of developing cancer^(^ ^5^ ^)^.

Much of the data used to generate these recommendations arose from associative relationships between single exposures and cancer incidence or mortality. However, cancer is likely caused by the interplay of many, rather than single, risk factors and therefore it is important to assess the integrated effect of multiple cancer risk factors^(^ ^6^ ^)^. Complex health indices that reflect many processes occurring simultaneously have been widely used to assess the impact of following health recommendations or guidelines on health outcomes^(^ ^7^ ^–^ ^9^ ^)^. A number of recent studies have used such indices to assess the association between following cancer-specific recommendations and subsequent cancer risk, and have generally^(^ ^6^ ^,^ ^10^ ^–^ ^14^ ^)^, but not always^(^ ^15^ ^)^, shown protective effects. However, lifestyle behaviours vary among populations^(^ ^16^ ^)^ and therefore results from some populations may not generalize to all populations.

In our previous study, we investigated adherence to WCRF/AICR recommendations reported by participants in Alberta’s Tomorrow Project (ATP) by creating a composite score^(^ ^17^ ^)^. The overall adherence to these cancer-specific recommendations in this cohort was low (60 % of participants met ≤3 recommendations), although women reported better adherence than men. The low level of adherence to WCRF/AICR recommendations reported in the ATP cohort may be partly caused by unawareness of these cancer-specific recommendations. Lack of concrete evidence of a beneficial effect of following these recommendations on cancer risk in this population may also be an important factor for the observed poor adherence. Hence, the aim of the present study was to extend the previous analysis to assess the association between adherence to WCRF/AICR recommendations, based on a composite score, and subsequent risk of developing cancer in ATP, a prospective cohort study.

## Methods

### Study population

The present study is based on data collected from participants in ATP. Recruitment methods have been reported in detail previously^(^ ^18^ ^,^ ^19^ ^)^ and are presented in brief here. From 2001 to 2009, 31 208 adults living in Alberta, Canada were enrolled into ATP by random digit dialling, which facilitated balanced recruitment across the province. Eligible participants were mailed a consent form and a Health and Lifestyle Questionnaire (HLQ), followed by a Canadian Diet History Questionnaire (C-DHQ)^(^ ^20^ ^)^ and a Past-Year Total Physical Activity Questionnaire (PYTPAQ)^(^ ^21^ ^)^. Participants also consented to linkage with the Alberta Cancer Registry (ACR). For the purpose of the current analysis, participants who were recruited as ‘second in household’ (*n* 382), outside the 35–69 year age range at the time of completing the HLQ (*n* 50), pregnant women (*n* 65), those who had BMI<18·5 kg/m^2^ (*n* 220, to minimize the potential influence of pre-existing conditions), personal history of cancer other than non-melanoma skin cancer prior to enrolment (*n* 81) and who reported implausible energy intakes assessed by the C-DHQ (<3347 or >17 573 kJ/d (<800 or >4200 kcal/d) for men and <2510 or >14 644 kJ/d (<600 or >3500 kcal/d) for women; *n* 1013)^(^ ^22^ ^)^ were excluded. Participants were also excluded if their log-transformed total energy expenditure derived from the PYTPAQ fell outside two interquartile ranges from the first and third quartile cut-offs^(^ ^23^ ^,^ ^24^ ^)^ (*n* 60). Finally, participants who were not living in Alberta at the time of enrolment (precluding linkage with the ACR for cancer case identification; *n* 28) and who did not complete the C-DHQ or PYTPAQ (*n* 4209) were also excluded. A final sample of 25 100 adults was included in the analysis.

### Cancer prevention recommendations adherence score

The WCRF/AICR recommendations include eight general recommendations, presented as ‘public health goals’ and ‘personal recommendations’, and two special recommendations pertaining specifically to breast-feeding and to cancer survivors. In our previous study, we constructed a composite score to assess the combined impacts of lifestyle factors based on adherence to WCRF/AICR personal recommendations for cancer prevention^(^ ^17^ ^)^. In that study, body fatness, physical activity, fruit and vegetable consumption, red meat consumption, alcoholic drinks and the intake of dietary supplements were selected as the component variables to form a modified composite index to study the combined impacts of lifestyle factors on subsequent cancer risk (Table 1). Adherence to the recommendation for dietary supplement use was not operationalized in many previous studies due to unavailability of data or the consideration of reported beneficial effects for certain long-term health conditions^(^ ^25^ ^–^ ^27^ ^)^. Despite these positive aspects, there is insufficient evidence to support the use of dietary supplements in cancer prevention^(^ ^28^ ^,^ ^29^ ^)^. In addition, some dietary supplements have been reported to increase cancer risk in some populations^(^ ^30^ ^,^ ^31^ ^)^. Hence the WCRF/AICR suggest avoiding dietary supplements for cancer prevention, stating that required nutrients should be obtained through consumption of whole foods instead^(^ ^5^ ^)^, and therefore this variable was included in the composite score. Tobacco exposure is one of the most well-defined cancer risk factors^(^ ^32^ ^,^ ^33^ ^)^, but is not part of the WCRF/AICR diet, nutrition and physical activity recommendations for cancer prevention, and therefore it was removed from the composite score in contrast to our previous report^(^ ^17^ ^)^. Instead, tobacco exposure was treated here as an adjusting covariate in the statistical models. Finally, three WCRF/AICR recommendations (‘foods and drinks that promote weight gain’, ‘preservation, processing, preparation’ and ‘breast-feeding’) were not included in the scoring because this information was not available from the questionnaires and/or it was not possible to quantify the adherence. The special recommendation for cancer survivors was also not included in the scoring because the current analysis was focused on participants’ adherence to recommendations prior to a cancer diagnosis.

**Table 1:** Proportions of Alberta’s Tomorrow Project participants meeting selected WCRF/AICR recommendations

WCRF/AICR recommendation	Operationalization	Scoring*	All (%)	Men (%)	Women (%)
Body fatness	BMI≥25·0 kg/m^2^	0			
	BMI<25·0 kg/m^2^	1	33·8	23·0	40·3
Physical activity	<210 min of moderate/vigorous-intensity† recreational physical activity/week over the last 12 months	0			
	≥210 min of moderate/vigorous-intensity recreational physical activity/week over the last 12 months	1	48·1	51·0	46·4
Plant foods	<5 servings of fruit and vegetables/d over the past 12 months‡	0			
	≥5 servings of fruit and vegetables/d over the past 12 months	1	38·9	32·8	42·5
Animal foods	≥500 g of red meat/week§	0			
	<500 g of red meat/week	1	80·1	64·7	89·3
Alcoholic drinks	>2 drinks/d for men and >1 drink/d for women	0			
	≤2 drinks/d for men and ≤1 drink/d for women	1	87·9	87·6	88·0
Dietary supplements	At least one dietary supplement over the past 12 months║	0			
	No dietary supplement over the past 12 months	1	19·6	28·7	14·2

WCRF/AICR, World Cancer Research Fund/American Institute for Cancer Research.

* Participants received a score of 1 if they met the recommendation and 0 if they did not.

† Calculated by metabolic equivalent of task (MET) values obtained from data reported on the Past-Year Total Physical Activity Questionnaire; MET≥3 was considered the cut-off reflecting moderate/vigorous-intensity physical activity.

‡ Excluded dry beans and peas, white potato, starchy vegetables, fruit juice and fruit drinks. Data were generated by Diet*Calc software based on the food frequency Canadian Diet History Questionnaire (CDHQ).

§ Included beef, lamb and pork; excluded organ meats.

║ Included vitamin A, thiamin, riboflavin, niacin, vitamin B_6_, folic acid, vitamin B_12_, vitamin C, vitamin D, vitamin E, β-carotene, Ca, Mg, Fe, Zn, Cu and Se. Supplement use was assessed by the CDHQ.

For each component variable, participants were assigned 1 point if they met the recommendation and 0 if they did not. The individual points were summed to one single adherence composite score for each participant, to a maximum of 6. Responses to the HLQ, C-DHQ (calculated by Diet*Calc software version 1.4.2; National Cancer Institute) and PYTPAQ questionnaires were used to determine adherence to the selected WCRF/AICR recommendations as follows.

1. Body fatness: self-reported height and weight, obtained from the HLQ, were used to obtain BMI (calculated as weight (in kilograms) divided by the square of height (in metres)), which was selected to be the indicator of body fatness; BMI<25·0 kg/m^2^ was defined as adherence to this recommendation.
2. Physical activity was assessed from the PYTPAQ, which collected information on recreational, occupational, transportation and household activities over the past year. A total of ≥210 min of moderate- and vigorous-intensity recreational activities per week was used to indicate adherence to this recommendation.
3. Fruit and vegetable consumption was assessed from the C-DHQ, which is a 124-item FFQ that assessed past-year intake of foods, beverages and dietary supplements; consuming ≥5 servings of fruit and vegetables combined (excluding dry beans and peas, white potato, starchy vegetables, fruit juice and fruit drinks) per day was defined as meeting the recommendation.
4. Red meat consumption was assessed from the C-DHQ; intake of <500 g of red meat per week, including beef, lamb and pork, was defined as meeting the recommendation.
5. Alcohol usage was extracted from the C-DHQ and is the only sex-based recommendation adopted in the present study; consuming ≤2 drinks per day for men and ≤1 drink per day for women was defined as meeting the recommendation.
6. Dietary supplements use was assessed using the C-DHQ; no reported diet supplement use was considered as adherence to this recommendation.

### Incidence of cancer

The primary outcome in the present study was any malignant cancer incidence identified via linkage with the ACR. Non-melanoma skin cancer was not included in the analysis due to its curable nature and inconsistent coding across registries. The ACR uses the International Classification of Diseases (ICD) for Oncology coding system (3rd edition: ICD-O-3) to classify cancers by site (topography) and histology (morphology), and collaborative staging rules are used to stage cancers^(^ ^34^ ^,^ ^35^ ^)^. The ACR has Gold Standard Certification by the North American Association of Central Cancer Registries for achieving the highest standard (case ascertainment is consistently above 95 %) for completeness, accuracy and timely data entry^(^ ^36^ ^)^. In addition to the four most prevalent individual cancer types (breast, prostate, colorectal and lung) reported in ATP participants, bladder, colon, oesophagus, kidney, larynx, liver, lung and bronchus, ovary (mucinous tumours), pancreas, rectum, stomach and uterine cervical cancers were further grouped as ‘smoking-related cancers’^(^ ^37^ ^,^ ^38^ ^)^. Breast, prostate, colorectal, endometrial, kidney and ovarian cancers were grouped as ‘obesity-related cancers’^(^ ^15^ ^)^. The date of completed HLQ was taken as the starting time point (baseline) and the date of cancer diagnosis (via the ACR) or censoring (10 November 2016) was considered as the ending time point (follow-up).

### Statistical analysis

The outcome and baseline characteristics of participants are presented as means and sd for continuous variables, and as counts and percentages for categorical variables.

Multivariable Cox proportional hazard regression models were employed to investigate the association between cancer outcomes and adherence scores (composite score as well as individual component scores). The proportional hazard assumption was tested graphically and no significant deviation from proportionality was observed. The clinical outcomes assessed in the present study include all cancer types combined, the four most prevalent individual cancer sites (listed above), smoking-related cancers and obesity-related cancers. Given previous reports that most modifiable risk factors may have limited association with prostate cancer risk^(^ ^5^ ^,^ ^39^ ^–^ ^41^ ^)^, ‘all cancer types excluding prostate cancer’ was also set as a clinical outcome to assess the impact of including/excluding prostate cancer in the composite cancer outcome. The composite adherence score was the major predictor variable in the estimation models. Survival analysis was also conducted on the composite adherence score categorized from low to high: category 1 (C1; score=0–2), category 2 (C2; score=3) and category 3 (C3; score=4–6). The categories were determined by the score distribution and with consideration for the sample sizes and the numbers of cases at each category level. The lowest category (C1) was treated as the reference level. Trends in associations were tested by modelling the constructed adherence score as an ordinal variable from 1 to 5 (1=0–1; 2=2; 3=3; 4=4; 5=5–6). The reported estimations were fully adjusted using age at baseline (continuous in years; removing this covariate when age was treated as primary time variable in the survival model), sex (only in the sex combined model), marital status (living with partner; living without partner), education level (high school or lower; college; university), employment status (not employed; retired; employed part-time; employed full-time), annual household income (<$CAN 70 000; ≥$CAN 70 000), tobacco exposure (current daily and occasional smokers; former daily smokers; and participants who were exposed to second-hand smoke on most days in the past year were coded as ‘yes’ for tobacco exposure, otherwise ‘no’), first-degree family history of cancer (yes; no) and personal history of chronic disease (yes; no), as well as use of hormone replacement therapy in women (yes; no). The estimations from survival analyses were reported as hazard ratios (HR) and corresponding 95 % CI.

Given the uncertainty around use of dietary supplements for cancer prevention, and the influence of tobacco exposure on cancer risk despite its exclusion from the WCRF/AICR diet and physical activity recommendations, sensitivity analyses were conducted to compare the impact of including/excluding dietary supplements as well as tobacco exposure, separately, in the composite adherence score on the association with cancer risk. Finally, selection of time scales in Cox regression models has been suggested to have some impact on estimations of the effect of time-varying environmental exposures^(^ ^42^ ^)^. Therefore, while time duration (from the date of receipt of HLQ to the date of diagnosis/censoring) was used as the primary time scale in the survival models, a sensitivity analysis was also conducted using age at diagnosis/censoring as the time variable. Additional sensitivity analyses were performed for physical activity (due to differences in international and national recommendations for physical activity of 210 *v*. 150 min/week, a cut-point of 150 min moderate/vigorous-intensity physical activity per week was analysed) and a basic Cox model with adjustment only for age and tobacco exposure (to determine the influence of including additional variables in the full model).

The statistical software package SAS version 9.2 of the SAS System for Linux was employed for all analyses and all statistical tests were set as two-sided.

## Results

Among the 25 100 participants in the present study, 63 % were women and the mean age at baseline was 50·5 (sd 9·2) years. Overall, in this cohort, women reported higher adherence scores than men, except for adherence to physical activity and dietary supplement recommendations (Table 1). Baseline sociodemographic and health characteristics of participants stratified by sex and categories of adherence to WCRF/AICR recommendations score are presented in Table 2. A greater proportion of men than women were employed full-time. Over half of participants reported a first-degree family history of cancer and approximately 45 % of participants also reported a personal history of chronic disease. In addition, about 34 % of women reported using hormone replacement therapy.

**Table 2:** Baseline characteristics of Alberta’s Tomorrow Project participants stratified by categories reflecting low to high adherence to WCRF/AICR recommendations

Baseline characteristic/category	All participants	Men			Women		
WCRF/AICR score category	All (0–6)	C1 (0–2)	C2 (3)	C3 (4–6)	C1 (0–2)	C2 (3)	C3 (4–6)
No. of participants (*n* *)	25 100	3455	3262	2596	4105	5631	6051
%	100·0	37·1	35·0	27·9	26·0	35·7	38·3
Age (years), mean	50·5	50·5	50·7	50·5	51·3	50·8	49·4
sd	9·2	8·9	9·1	9·3	9·1	9·3	9·1
Marital status† (%‡)							
Living with partner	78·9	83·4	83·4	83·5	75·4	75·8	77·0
Education level§ (%‡)							
High school or lower	27·5	28·8	24·6	18·6	35·5	30·7	24·0
College	39·5	42·8	40·9	36·9	39·4	39·9	37·9
University	33·0	28·4	34·6	44·6	25·1	29·5	38·1
Employment status║ (%‡)							
Not employed	13·7	6·2	5·3	4·6	19·0	18·7	18·1
Retired	13·5	10·6	13·4	14·9	14·3	14·5	13·4
Employed part-time	17·0	6·5	6·6	6·5	21·6	22·7	24·4
Employed full-time	55·8	76·7	74·7	74·0	45·1	44·1	44·1
Annual household income (%‡)							
≥CAN $70 000	48·9	54·1	55·4	58·5	38·4	42·5	51·2
Tobacco exposure¶ (%‡)							
Exposed to tobacco in the past year	58·7	67·5	63·4	53·9	63·7	57·7	50·7
First-degree family history of cancer** (%‡)							
Yes	53·4	51·5	51·2	50·1	56·6	55·0	53·4
Personal history of chronic disease†† (%‡)							
Yes	45·1	52·4	49·4	44·1	51·7	44·4	35·4
HRT‡‡ (%‡)							
Yes	N/A				37·6	35·0	31·4

WCRF/AICR, World Cancer Research Fund/American Institute for Cancer Research; C1–3, category 1–3; HRT, hormone replacement therapy; N/A, not applicable.

* Values describing number of participants in each category reflect row percentages (prevalence of men and women in each category as a percentage of the total number of men or women).

† Living without partner defined as divorced, separated, widowed or single (never married); living with partner defined as married, or not married but living with someone.

‡ Values in each cell reflect column percentages within each category.

§ High school or lower defined as did not complete Grade 8, completed Grade 8 but not high school, or completed high school; college defined as some technical school/college training completed, or completed technical school/college training; university defined as some part of university degree completed, completed university degree, some part of postgraduate university degree completed, or completed university postgraduate degree.

║ Not employed defined as not employed but looking for work, homemaker or student; employed part-time defined as working less than 30 h/week; employed full-time defined as working 30 h/week or more.

¶ Included daily smokers, current occasional smokers, former daily smokers and participants who were exposed to second-hand smoke on most days in the past year.

** First-degree family history of cancer defined as any one of father, mother, brother, sister, son, daughter of the participant had been diagnosed with cancer; otherwise ‘no’.

†† Personal history of chronic disease defined as participant reported having any one of the following medical conditions: high blood pressure, angina, high cholesterol, heart attack, stroke, emphysema, chronic bronchitis, diabetes, ulcerative colitis, Crohn’s disease, hepatitis, liver cirrhosis; otherwise ‘no’.

‡‡ HRT defined as women reported being on hormone replacement therapy; otherwise ‘no’.

With a mean follow-up of 11·7 (sd 3·0) years (total of 293 329 person-years), 2066 incident cancer cases were identified. The distribution of incident cancer cases according to the composite adherence score is presented in Table 3. Breast, prostate, colorectal and lung were the most common cancer sites, accounting for over half of total cancer cases.

**Table 3:** Frequency of individual cancer sites and distribution of all cancer sites combined according to the WCFR/AICR composite adherence score* in Alberta’s Tomorrow Project participants

	All participants		Men		Women	
Cancer site†/composite score	*n*	%	*n*	%	*n*	%
Breast cancer	454	20·8	N/A		454	35·8
Prostate cancer	360	16·5	360	39·3	N/A	
Colorectal cancer	221	10·1	103	11·3	118	9·3
Lung and bronchus cancer	186	8·5	65	7·1	121	9·5
Other cancer‡	962	44·1	387	42·3	575	45·3
All cancer§	2066	100	860	100	1206	100
Adherence to WCRF/AICR recommendations composite score						
0	8	0·4	5	0·6	3	0·3
1	112	5·4	73	8·5	39	3·2
2	556	26·9	247	28·7	309	25·6
3	776	37·6	307	35·7	469	38·9
4	473	22·9	180	20·9	293	24·3
5	126	6·1	38	4·4	88	7·3
6	15	0·7	10	1·2	5	0·4

WCRF/AICR, World Cancer Research Fund/American Institute for Cancer Research; N/A, not applicable.

* Composite adherence score is based on six WCRF/AICR lifestyle recommendations.

† Only invasive cancers reported by the Alberta Cancer Registry were included in the present study. Non-melanoma skin cancers were excluded.

‡ Other cancers included bladder, brain, cervix, oesophagus, kidney, larynx, leukaemia, liver, lymphoma, non-Hodgkin lymphoma, ovary, pancreas, stomach, thyroid, trachea, uterus and others not specified.

§ The numbers reported in this row are less than the sum of the above listed individual/cluster of cancers due to the repeated counts of multiple cancer types for individual participants (i.e. ‘all cancer’ was defined as the incidence of any cancer, with each participant only counted once regardless of incidence of multiple cancer sites).

HR for the risk of developing cancer across adherence score categories are presented in Table 4. After adjusting for potential confounding factors, participants who reported being most adherent to the six selected WCRF/AICR recommendations (C3: 4–6 recommendations met) were 13 % (HR=0·87; 95 % CI 0·78, 0·98) less likely to develop cancer (all cancers combined) compared with those who were the least adherent (C1: 0–2 recommendations met). Among the full list of covariates, tobacco exposure (HR=0·82; 95 % CI 0·75, 0·90) was one of the most significant adjustments in the full model. An inverse association was also observed for colorectal cancer and obesity-related cancers. Furthermore, each one unit increase in the adherence score (i.e. each additional recommendation met) corresponded to a 5 % (HR=0·95; 95 % CI 0·91, 0·99) reduction in risk of developing cancer (see online supplementary material, Supplemental Table 1). When stratified by sex, similar results were observed for women, but not men.

**Table 4:** Associations between categories of the WCRF/AICR adherence score and risk of cancer in Alberta’s Tomorrow Project participants

	All participants (*n* 25 100)		Men (*n* 9313)		Women (*n* 15 787)	
Cluster of cancer outcomes/adherence score category	HR*	95 % CI	HR*	95 % CI	HR*	95 % CI
All cancer (*n*_cases_: all participants=2066; men=860; women=1206)						
C1 (0–2)	1·00	Ref.	1·00	Ref.	1·00	Ref.
C2 (3)	0·99	0·89, 1·10	0·97	0·83, 1·14	0·99	0·87, 1·15
C3 (4–6)	0·87	0·78, 0·98	0·92	0·78, 1·09	0·83	0·72, 0·96
*P* trend†	<0·001		0·453		<0·001	
Breast cancer (*n*_cases_: 454)						
C1 (0–2)					1·00	Ref.
C2 (3)					1·07	0·85, 1·35
C3 (4–6)					0·86	0·68, 1·09
*P* trend†					0·048	
Prostate cancer (*n*_cases_: 360)						
C1 (0–2)			1·00	Ref.		
C2 (3)			0·96	0·75, 1·23		
C3 (4–6)			0·99	0·76, 1·29		
*P* trend†			0·622			
Colorectal cancer (*n*_cases_: all participants=221; men=103; women=118)						
C1 (0–2)	1·00	Ref.	1·00	Ref.	1·00	Ref.
C2 (3)	0·86	0·63, 1·16	0·90	0·58, 1·41	0·83	0·54, 1·26
C3 (4–6)	0·67	0·48, 0·95	0·82	0·49, 1·36	0·58	0·36, 0·94
*P* trend†	0·002		0·332		0·006	
Lung cancer (*n*_cases_: all participants=186; men=65; women=121)						
C1 (0–2)	1·00	Ref.	1·00	Ref.	1·00	Ref.
C2 (3)	0·95	0·68, 1·34	0·70	0·40, 1·23	1·15	0·75, 1·77
C3 (4–6)	0·84	0·58, 1·22	0·68	0·36, 1·30	0·96	0·60, 1·55
*P* trend†	0·012		0·035		0·089	
All cancer excluding prostate cancer (*n*_cases_: all participants=1747; men=541)						
C1 (0–2)	1·00	Ref.	1·00	Ref.		
C2 (3)	0·98	0·87, 1·09	0·93	0·77, 1·13		
C3 (4–6)	0·85	0·76, 0·96	0·88	0·71, 1·10		
*P* trend†	<0·001		0·144			
Smoking-related cancer‡ (*n*_cases_: all participants=611; men=288; women=323)						
C1 (0–2)	1·00	Ref.	1·00	Ref.	1·00	Ref.
C2 (3)	0·91	0·75, 1·09	0·80	0·61, 1·04	1·03	0·80, 1·35
C3 (4–6)	0·79	0·65, 0·97	0·78	0·58, 1·05	0·84	0·63, 1·12
*P* trend†	<0·001		0·007		0·002	
Obesity-related cancer§ (*n*_cases_: all participants=1209; men=470; women=739)						
C1 (0–2)	1·00	Ref.	1·00	Ref.	1·00	Ref.
C2 (3)	0·99	0·87, 1·14	0·94	0·76, 1·16	1·01	0·85, 1·21
C3 (4–6)	0·86	0·75, 0·99	0·94	0·75, 1·18	0·79	0·66, 0·96
*P* trend†	0·002		0·971		<0·001	

WCRF/AICR, World Cancer Research Fund/American Institute for Cancer Research; HR, hazard ratio; C1–3, category 1–3; Ref., reference category.

* HR were estimated using a Cox regression model adjusted for age (continuous in years), sex (in sex combined model), marital status (living without partner, living with partner), education level (high school or lower, college, university), employment status (not employed, retired, employed part-time, employed full-time), annual household income (<$CAN 70 000, ≥$CAN 70 000), tobacco exposure (no, yes), first-degree family history of cancer (no, yes) and personal history of chronic disease (no, yes for following conditions: high blood pressure, angina, high cholesterol in blood, heart attack, stroke, emphysema, chronic bronchitis, diabetes, ulcerative colitis, Crohn’s disease, hepatitis, liver cirrhosis), as well as hormone replacement therapy in women.

† Trend tested by modelling categories of adherence to the WCRF/AICR composite score as an ordinal variable from 1 to 5 (1=0–1; 2=2; 3=3; 4=4; 5=5–6).

‡ Smoking-related cancers included bladder, colon, oesophagus, kidney, larynx, liver, lung and bronchus, ovary (mucinous tumours), pancreas, rectum, stomach and uterine cervical cancers.

§ Obesity-related cancers included breast, prostate, colon, rectum, endometrial, kidney and ovarian cancers.

**Fig. 1:**
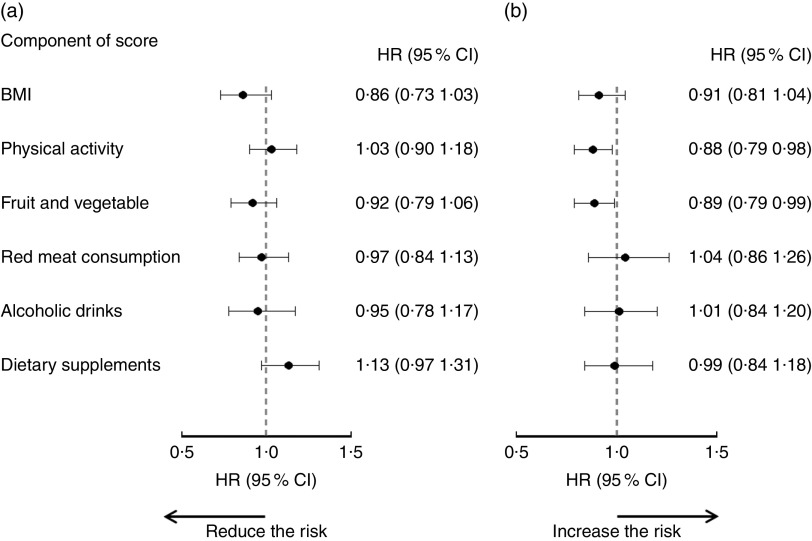
Associations between adherence to individual World Cancer Research Fund (WCRF)/American Institute for Cancer Research (AICR) recommendations and risk of cancer in Alberta’s Tomorrow Project participants, stratified by sex: (a) men (860 cases/9313); (b) women (1206 cases/15 787). Hazard ratios (HR), with their 95 % CI represented by horizontal bars, were estimated by comparing those who met individual WCRF/AICR recommendations (score=1) with those who did not (score=0; reference level). In addition to the mutual adjustment of the individual component scores, HR were also adjusted for age (continuous in years), marital status (live without partner, live with partner), education level (high school or lower, college, university), employment status (not employed, retired, employed part-time, employed full-time), annual household income (<$CAN 70 000, ≥$CAN 70 000), tobacco exposure (no, yes), first-degree family history of cancer (no, yes) and personal history of chronic disease (no, yes for following conditions: high blood pressure, angina, high cholesterol in blood, heart attack, stroke, emphysema, chronic bronchitis, diabetes, ulcerative colitis, Crohn’s disease, hepatitis and liver cirrhosis), as well as use of hormone replacement therapy in women

The associations between adherence to each individual recommendation and risk of any cancer are illustrated in Fig. 1. From point estimations, the magnitude of associations varied among the individual WCRF/AICR recommendations. None of the individual recommendations was associated with reduced cancer risk in men. In women, meeting physical activity (HR=0·88; 95 % CI 0·79, 0·98) and fruit and vegetable (HR=0·89; 95 % CI 0·79, 0·99) recommendations were associated with lower risk of cancer (all cancers combined). No significant impact was observed when the use of dietary supplements was removed from the composite adherence score (see online supplementary material, Supplemental Table 1).

Including tobacco exposure in the adherence score, instead of adjusting for it in the survival model, did not change the overall patterns (inverse association and sex-based difference) of associations observed, but increased the magnitude of the associations (see online supplementary material, Supplemental Table 1). Excluding the dietary supplements from the adherence score and basic Cox model with adjustment only for age and tobacco also did not change the observed inverse associations. Finally, age at diagnosis has been used as the primary time scale in some epidemiological studies due to the extension of some exposure risk factors beyond the time of entry^(^ ^42^ ^)^; however, no noteworthy differences in associations (in either magnitude or direction) were observed when changing the time scale in the regression analysis (Supplemental Table 1), indicating that the effect of changing the primary time variable in the Cox models was negligible.

Further subgroup analyses were conducted to determine if following recommendations was as effective at reducing cancer risk in participants with pre-existing health conditions or chronic disease at enrolment compared with those without (Table 5). The previously observed inverse associations (Table 4) were found only in participants without chronic conditions at baseline (corresponding to a 7 % reduction in cancer risk with each additional recommendation met). In participants with existing chronic conditions at baseline, no associations between adherence to recommendations and cancer risk were observed in all participants, or separately in men and women (Table 5).

**Table 5:** Subgroup analysis of the association between WCRF/AICR recommendation adherence composite score and risk of any cancer in participants with and without baseline chronic conditions in Alberta’s Tomorrow Project participants

Baseline health condition/participants	No. of cancer cases	HR*	95 % CI
With chronic conditions† at baseline			
All (*n* 11 330)	1165	0·97	0·92, 1·03
Men (*n* 4564)	523	0·96	0·88, 1·04
Women (*n* 6766)	642	0·98	0·91, 1·06
Without chronic conditions† at baseline			
All (*n* 13 770)	901	0·93	0·87, 0·98
Men (*n* 4749)	337	1·01	0·92, 1·12
Women (*n* 9021)	564	0·87	0·80, 0·94
With high blood pressure or high cholesterol or diabetes at baseline			
All (*n* 10 162)	1049	0·98	0·92, 1·04
Men (*n* 4176)	472	0·97	0·89, 1·05
Women (*n* 5986)	577	0·99	0·91, 1·07
Without high blood pressure or high cholesterol or diabetes at baseline			
All (*n* 14 938)	1017	0·92	0·87, 0·98
Men (*n* 5137)	388	0·99	0·91, 1·09
Women (*n* 9801)	629	0·87	0·81, 0·94

WCRF/AICR, World Cancer Research Fund/American Institute for Cancer Research; HR, hazard ratio.

* HR were estimated using a Cox regression model by each one additional recommendation met, adjusted for age (continuous in years), sex (only in sex combined model), marital status (living without partner, living with partner), education level (high school or lower, college, university), employment status (not employed, retired, employed part-time, employed full-time), annual household income (<$CAN 70 000, ≥$CAN 70 000), first-degree family history of cancer (no, yes), as well as hormone replacement therapy in women.

† If participants reported having a personal history of any one of the following medical conditions: high blood pressure, angina, high cholesterol in blood, heart attack, stroke, emphysema, chronic bronchitis, diabetes, ulcerative colitis, Crohn’s disease, hepatitis or liver cirrhosis.

## Discussion

In the present study we observed that greater adherence to six WCRF/AICR lifestyle recommendations for cancer prevention was associated with lower risk of cancer in this cohort. This observation is consistent with previous reports^(^ ^10^ ^–^ ^12^ ^)^, as well as a recently published systematic review^(^ ^43^ ^)^, suggesting encouraging greater adherence to recommendations related to modifiable lifestyle behaviours as an effective population health strategy to reduce cancer risk.

The inverse associations found between adherence to the WCRF/AICR recommendations and cancer risk were observed mainly in women but not in men in the present study, for groupings of different cancers, as well as individually for colorectal cancer. Even though sex-specific differences are well documented in many disorders, the sex-based difference observed in the present study has not been consistently reported in similar studies, possibly because: (i) sex was treated only as a confounding factor in the estimation models^(^ ^13^ ^,^ ^15^ ^)^; (ii) the analysis was performed in a single-sex cohort^(^ ^44^ ^,^ ^45^ ^)^; or (iii) limited available sample size, which may lead to lack of observed effect between adherence to WCRF/AICR recommendations and cancer risk^(^ ^15^ ^)^. Indeed, a larger sample size in women than in men may explain some of the differences in associations observed here between men and women. However, a prospective Danish cohort study observed a significant association between adherence to lifestyle recommendations and risk of colorectal cancer in men but not women^(^ ^46^ ^)^, although both the component selection of the lifestyle index and operationalization criteria differed from our study and thus it is challenging to compare the results directly. In a study from the European Prospective Investigation into Cancer and Nutrition (EPIC) cohort, the association between adherence to WCRF/AICR recommendations and reduced cancer risk was observed in both men and women^(^ ^10^ ^)^. Adherence to the American Cancer Society (ACS) cancer prevention guidelines was also reported to be associated with a reduction in all-cancer incidence in both men and women in the NIH–AARP Diet and Health Study^(^ ^12^ ^)^. However, in the NIH–AARP study, in addition to the more senior age (participants were 50–71 years old at recruitment), the association observed in men (HR=0·90; 95 % CI 0·87, 0·93) was weaker than that observed in women (HR=0·81; 95 % CI 0·77, 0·84). Neither study observed associations between recommendation scores and risk of prostate cancer. These results are not unexpected, given some reports that most modifiable risk factors have a less protective effect for prostate cancer than for other cancer types^(^ ^5^ ^,^ ^39^ ^,^ ^40^ ^)^. This is in agreement with our observation that the association between cancer risk and adherence to prevention recommendations in men became stronger when prostate cancer cases were removed from the total cancer outcomes.

In the present study, no associations with cancer risk were detected in men for any single component of the composite score, whereas in women two recommendations (physical activity and fruit and vegetable consumption) were associated with reduced cancer risk. This difference in the relationship of individual components with overall cancer risk may be a factor contributing to the sex-based difference observed in our study. For example, use of the same cut-off values for recommendations for both sexes (except for alcohol consumption), together with the sex-based differences in associations observed herein, suggest that the underlying assumption of a similar dose–response curve for cancer protection for both men and women may require further exploration^(^ ^47^ ^)^. Apart from the epidemiological and statistical factors above, the biological variation, i.e. sex-specific hormone and endocrine fluctuation, between men and women should also be considered. Indeed, research into sex differences drew attention because some adverse effects were found only in women in some clinical trials which were designed based on the results from single-sex preclinical research (predominantly male, to avoid the hormonal variations in female animal models). As a result, the National Institutes of Health (NIH) has implemented new policy to account for participants’ sex as a biological variable in NIH-funded research. Further studies into the sex-specific differences observed in the present study may identify opportunities to develop sex-based recommendations to reduce cancer risk both in men and women.

Selecting appropriate indicators to represent the component of a composite score is challenging. For example, BMI within the normal range at the time of completing the enrolment questionnaire in the present study cannot be used to ascertain adherence to the other two WCRF/AICR personal recommendations pertaining to body fatness (‘ensure that body weight through childhood and adolescent growth projects towards the lower end of the normal BMI range at age 21’ and ‘avoid weight gain and increases in waist circumference throughout adulthood’). BMI may be not an appropriate indicator of body composition^(^ ^48^ ^,^ ^49^ ^)^ and other indicators, such as waist circumference or waist-to-hip ratio, should be explored in future studies. Further, to reflect the WCRF/AICR report, a cut-off of two alcoholic drinks per day for men and one drink for women was applied in the present study. However, the fact that ethanol has been classified as a class I carcinogen^(^ ^50^ ^)^ promotes a consideration of avoiding alcohol consumption completely for cancer prevention, and indeed the most recent evidence synthesis from the WCRF Continuous Update Project recommended completely abstaining from alcohol for cancer prevention^(^ ^51^ ^)^. Finally, even though some dietary supplements provide beneficial effects for certain health conditions^(^ ^25^ ^,^ ^27^ ^)^, the WCRF/AICR suggest avoiding dietary supplements for cancer prevention, stating that required nutrients should be obtained through consumption of whole foods instead. We examined including/excluding the dietary supplements component in the composite score and found no significant changes in the associations; therefore, this binary variable may need to be teased out more carefully in future studies. For example, the type and dose of dietary supplements used and the reason for consumption may need to be taken into consideration, assuming relevant data become available in the future.

We observed that the inverse associations between following six WCRF/AICR recommendations and cancer incidence were attenuated in those participants who reported having a chronic health condition at enrolment, even in women. In agreement with these findings, a recent study from the Southern Community Cohort reported that a score reflecting adherence to ACS guidelines was inversely associated with cancer risk only among participants without pre-existing chronic disease^(^ ^14^ ^)^. These results, together with the fact that the prevalence of chronic health conditions or diseases (other than cancer) is high in older populations (~45 % in this cohort), suggest that sub-populations, such as participants with diabetes, may require more specific or intensive interventions to reduce cancer risk.

To our knowledge, the present study is the first to assess the impact of adherence to a suite of cancer prevention recommendations on risk of overall and site-specific cancers in a Canadian population. In addition, the results also suggest a sex-based difference in associations; however, the effect differences are minor and therefore caution should be taken in the interpretation of these results and more research in this area is warranted. Strengths of the present study include a large sample size, random digit dialling-based recruitment, prospective study design and objective measurement of cancer outcomes via linkage with an accredited cancer registry. In addition, having one composite score that reflects overall adherence to six cancer-specific recommendations simultaneously as a major predictor in the survival model also increases the analysis power and avoids the problem of potential multicollinearity that can arise when forcing many individual components together into a model^(^ ^52^ ^)^.

Limitations of the present study are those common to cohort studies, including potential measurement errors from self-reported diet and physical activity questionnaires^(^ ^17^ ^)^. In addition, the equal weighting applied to each component in the composite adherence score may not be ideal, as the strength of individual components to reduce cancer risk may be different. Finally, even though we adjusted for potential confounding variables in our models, some unknown factors (e.g. the magnitude and duration of stress), which were not operationalized based on the WCRF/AICR recommendations, as well as other dietary components (not included in these personal recommendations) may also account for the observed associations; identification of such variables would allow for a more comprehensive model to improve the estimation efficiency and accuracy.

## Conclusion

The present study provides support for encouraging adherence to cancer prevention recommendations to reduce cancer risk. Further research is needed to explore and understand potential sex-based differences that may modify the relationship between lifestyle factors and cancer risk. Health policy makers could consider adopting and promoting evidence-based prevention strategies and systematic allocation of health-care resources to promote adherence to these recommendations.

## Supplementary material

For supplementary material accompanying this paper visit https://doi.org/10.1017/S1368980018002689. click here to view supplementary material
