# Supplementary material for: Impact of adherence to cancer-specific prevention recommendations on subsequent risk of cancer in participants in Alberta’s Tomorrow Project
Source: Public Health Nutr. 2018 Oct 22;22(2):235–45. doi: 10.1017/S1368980018002689 (PMC6390391; doi:10.1017/S1368980018002689)
Supplement: Supplementary file 1 [file S1368980018002689sup001.docx]

**Supplementary Table S1**

**The impact of selection of components of the WCRF/AICR^a^ adherence composite score and primary time scale on the estimation of association between the risk of cancer and adherence composite score in Alberta’s Tomorrow Project participants**

| **Changing score composition/primary time scale** | **All participants**  **(25,100)** | | **Men**  **(9,313)** | | **Women**  **(15,787)** | |
| --- | --- | --- | --- | --- | --- | --- |
|  | **HR^b^** | **95% CI** | **HR^b^** | **95% CI** | **HR^b^** | **95% CI** |
| Adherence composite score with time duration as primary time scale (reference) | 0.95 | 0.91-0.99 | 0.98 | 0.92-1.04 | 0.92 | 0.87-0.98 |
| Excluding dietary supplements from adherence composite score | 0.94 | 0.89-0.98 | 0.95 | 0.89-1.02 | 0.92 | 0.87-0.97 |
| Adding tobacco exposure into adherence composite score^c^ | 0.93 | 0.89-0.96 | 0.97 | 0.91-1.02 | 0.89 | 0.85-0.94 |
| 150 min moderate/vigorous intensity physical activity per week selected as cut-point for adherence | 0.95 | 0.91-0.99 | 0.98 | 0.92-1.05 | 0.92 | 0.88-0.98 |
| Age at diagnosis/censoring as primary time variable in Cox model^d^ | 0.96 | 0.92-0.99 | 0.98 | 0.92-1.03 | 0.94 | 0.88-0.99 |
| Basic Cox model with adjustment only for age and tobacco exposure | 0.95 | 0.91-0.99 | 0.98 | 0.92-1.04 | 0.92 | 0.87-0.98 |

^a^ WCRF/AICR: World Cancer Research Fund / American Institute for Cancer Research.

^b^ Hazard ratios were estimated using a Cox regression model by each one additional recommendation met, adjusted for age (continuous in years), sex (in all participants model only), marital status (living without partner, living with partner), education level (high school or lower, college, university), employment status (not employed, retired, employed part-time, employed full-time), annual household income (<$70,000, ≥70,000), tobacco exposure (no, yes), first degree family history of cancer (no, yes), and personal history of chronic disease (no, yes for the following conditions: high blood pressure, angina, colitis, Crohn’s disease, hepatitis, liver cirrhosis), as well as HRT in women.

^c^ Because it was included in the composite score in this model, tobacco exposure was removed from the list of adjusting factors.

^d^ Age was removed from the list of adjusting factors in this model.
